# DNA Methylation in Multiple Myeloma Is Weakly Associated with Gene Transcription

**DOI:** 10.1371/journal.pone.0052626

**Published:** 2012-12-20

**Authors:** Sungwon Jung, Seungchan Kim, Molly Gale, Irene Cherni, Rafael Fonseca, John Carpten, Bodour Salhia

**Affiliations:** 1 Integrated Cancer Genomics Division, Translational Genomics Research Institute, Phoenix, Arizona, United States of America; 2 Mayo Clinic, Scottsdale, Arizona, United States of America; University of North Carolina at Chapel Hill, United States of America

## Abstract

Previous studies have now demonstrated that both genic and global hypomethylation characterizes the multiple myeloma (MM) epigenome. Whether these methylation changes are associated with global and corresponding increases (or decreases) in transcriptional activity are poorly understood. The purpose of our current study was to correlate DNA methylation levels in MM to gene expression. We analyzed matching datasets generated by the GoldenGate methylation BeadArray and Affymetrix gene expression platforms in 193 MM samples. We subsequently utilized two independent statistical approaches to identify methylation-expression correlations. In the first approach, we used a linear correlation parameter by computing a Pearson correlation coefficient. In the second approach, we discretized samples into low and high methylation groups and then compared the gene expression differences between the groups. Only methylation of 2.1% and 25.3% of CpG sites on the methylation array correlated to gene expression by Pearson correlation or the discretization method, respectively. Among the genes with methylation-expression correlations were IGF1R, DLC1, p16, and IL17RB. In conclusion, DNA methylation may directly regulate relatively few genes and suggests that additional studies are needed to determine the effects of genome-wide methylation changes in MM.

## Introduction

Multiple myeloma (MM) is an incurable late-stage plasma cell malignancy which accounts for about 10% of all hematological cancers [1]. Extensive analyses of gene expression profiles, genomic copy number and whole genomic sequencing have provided valuable insights into the molecular basis of MM [1], [2], [3]. These studies have led to the identification of several genetic and molecular subtypes that are associated with unique clinical and prognostic features. About one-half of myeloma patients have recurrent immunoglobulin gene translocations, while the other half are hyperdiploid [4]. While cyclin D regulation appears to be an early event in myeloma, a variety of other secondary events such as chromosome 13 monosomy and amplification of chromosome 1q are also known to commonly occur [1], [2], [3].

In contrast to genetic characterizations, much less is known about epigenetic changes in MM. Epigenetic modifications constitute a number of complex and interdependent mechanisms that have become recognized as critical facets of cancer development and progression [5], [6]. The biochemical modifications that govern epigenetics are DNA methylation, and post-translational modifications of histone proteins [5], [7], [8]. About 80% of CpG sites in mammalian cells are methylated, but both the CpG sites and their degree of methylation are unevenly distributed in the genome [9], [10]. CpG dinucleotides are largely concentrated in small regions termed “CpG islands”, which are found in about 55% of human gene promoters [11]. CpG loci in promoter-associated CpG islands are usually (but not always) unmethylated [12]. Recently we conducted a study to assess differential CpG methylation at about 1,500 genic loci during MM progression by profiling CD138(+) normal plasma cells (NPC) and comparing them to CD138(+) plasma cells from monoclonal gammapothy of undetermined significance (MGUS), smoldering myeloma (SMM), and MM specimens [13]. We showed that the vast majority of differentially methylated genes were hypomethylated, and that the overall degree of hypomethylation progressively increased with tumor grade [13].

Presently, the precise role of methylation in regulating gene expression is unclear. For many years, methylation was believed to play a crucial role in repressing gene expression, perhaps by blocking the promoters at which activating transcription factors bind. Studies have shown that methylation near gene promoters varies considerably depending on cell type, with more methylation of promoters inversely correlating with low or no transcription [14], [15]. To explore the relationship between gene expression and DNA methylation in MM, we employed two different comparison methods. For these approaches we used DNA methylation data obtained with the GoldenGate BeadArray technology along with corresponding array-based gene expression data from 193 human MM samples. We then validated the methylation-expression associations of a few selected genes by bisulfite pyrosequencing and quantitative reverse transcriptase-PCR (qRT-PCR) in an independent cohort of 43 MM samples.

## Methods

### DNA Methylation and Gene Expression Analyses

We used matching gene expression and methylation datasets previously generated. The gene expression dataset was downloaded from the Multiple Myeloma Genomics Portal (MMGP; http://www.broadinstitute.org/mmgp) which was generated as part of the Multiple Myeloma Research Consortium (MMRC) Genomics Initiative. Samples included a mix of newly diagnosed and previously treated patients with MM and covered the spectrum of genomic alterations known for this disease. Gene expression data was generated using the Affymetrix U133 Plus 2.0 arrays and both data and sample annotation are available for download.

Methylation data was previously generated for 140 MM samples using the GoldenGate Methylation Cancer Panel I (Illumina) for direct measurement of DNA methylation at 1,505 CpG sites selected from 807 genes [13]. In the current study, we expanded the methylation analysis to include 53 additional samples totaling 193. Therefore a total of 193 samples had matching DNA methylation and gene expression data and were analyzed in the current study. Details on the methods of the GoldenGate methylation assay and data analysis can be found in our previous reference [13]. All 807 genes in the methylation platform were covered by the gene expression array platform.

### Correlating DNA Methylation to Expression Using Pearson Correlation

**Figure 1:**
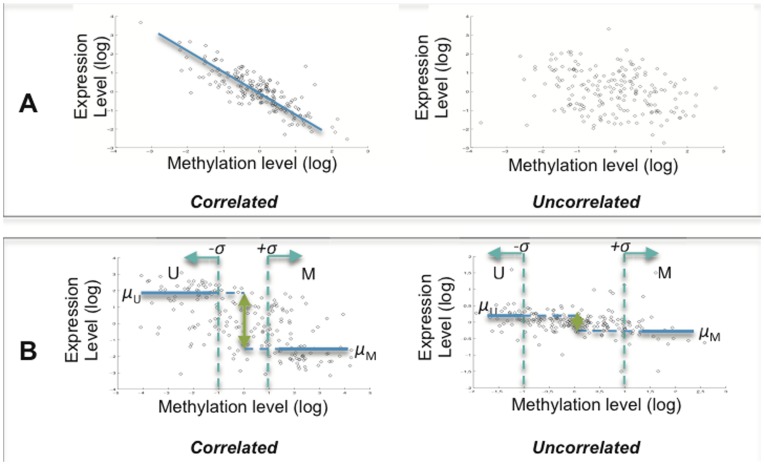
Methods used to identify genes with methylation-expression correlations. (A) Pearson correlation was used to measure linear relationships between DNA methylation and gene expression levels for 1505 CpG probes represented on the GoldenGate Methylation BeadArray. The panels represent examples of a gene with high (left) and low (right) Pearson correlation coefficients when analyzing DNA methylation levels (x axis) against gene expression levels (y axis). (B) A discretization approach was used to classify samples into *methylated* (M) or *unmethylated* (U) groups based on the mean (*μ*) methylation value and standard deviation (*σ*) of a given probe. Statistically significant gene expression differences between M and U groups indicated a methylation-expression correlation for the gene in question.

Both methylation and gene expression data from 193 MM samples were quantile-normalized to adjust technical variation between samples and distributions were standardized across genes and samples. A Pearson correlation coefficient (*ρ*) was computed for each methylation and gene expression probe pair, where both probes mapped to the same gene. A probe pair with *ρ*<−0.370 (representing 1% of the left tail of the Pearson correlation coefficient distribution) or *ρ*≥0.239 (1% right tail), and *P*<0.01 after 10,000 random permutations was considered significant (**Figure 1A**). Random permutation testing randomly shuffles the sample to sample mapping between expression data points and methylation data points and computes the probability that the observed correlation coefficient does not occur by random chance.

### Correlating DNA Methylation to Expression by Discretizing Samples into *methylated* or *unmethylated* Groups

Methylation and gene expression data from 193 MM samples were quantile-normalized and standardized across genes and samples. For each methylation probe with mean methylation level *μ* and standard deviation *σ*, samples were categorized into three different groups based on the distribution of values for each methylation probe across the sample set: *methylated* (M), *moderately methylated* or *unmethylated* (U). We therefore refer to this approach as “the discretization approach” because samples were discretized into one of the three groups. Samples with methylation levels ≥ *μ*+*σ* (mean plus one standard deviation) were classified into the *methylated* group; samples with methylation levels<*μ* – *σ* were classified as *unmethylated*; and all other remaining samples were considered *moderately methylated* samples for the given CpG locus interrogated by the probe and not considered further. Every probe in the methylation array had a gene expression probe that mapped to the same gene and was analyzed. Differential gene expression analysis comparing samples that were categorized as M and U was conducted with a *t*-test without assuming equal variance (Matlab software). If a gene was differentially expressed (*P*<0.05) between samples in the M and U groups, we considered CpG methylation to be correlated to expression of that gene.

### Class Enrichments for Samples Classified as *methylated* or *unmethylated*

Next we wanted to determine if samples in U and M groups had a tendency to harbor certain chromosomal gains/losses or belong to one of eight MM molecular subtypes based on the presence of translocations and cyclin D expression profiles (TC class). Chromosomal gains and losses, and TC class will be referred to as “*sample class*.” The common chromosomal abnormalities associated with MM considered in the sample class enrichment analysis included: hyperdiploid status, 1q amplification, 13 monosomy, and 17p deletions [16]. The chromosomal status of each sample was extrapolated from previously generated array-based comparative genomic hybridization (aCGH) data as previously described [13] and can also be downloaded from the MMGP. In addition, a patient’s TC class was also used for sample class enrichments. TC class is a gene expression-based molecular classification system, which is used to identify all the major cytogenetic categories in MM [4], [17]. These include: t(4;14)(p16;q32) (based on FGFR3/MMSET expression; referred to as 4p16); t(14;16)(q32;q23) and t(14;20) (based on expression of ITGB7 and MAF, referred to as Maf); t(6;14) assessed by high level of CCND3 expression (referred to as 6p21); t(11;14)(q13;q32) based on high level CCND1 expression (referred to 11q13); hyperdiploid MM based on aberrant high CCND1 expression with either low (referred to as D1) or high CCND2 expression (referred to as D1+D2 group); and hyperdiploid MM with low CCND1 but high CCND2 expression (referred to as D2). Presence of the t(4;14), t(14;20) translocations, 17p deletion and 13 monosomy are associated with high risk MM [16]. The absence of high risk features and presence of hyperdiploidy, and t(11;14) or t(6;14) translocations are associated with good risk MM [16].

To perform the class enrichment analysis, we considered only probes that had a statistically significant association with gene expression by our discretization approach. First the frequency of a *sample class* in U and M groups was determined to measure how common a *class* is in each group. Statistical significance was evaluated by performing 10 000 random permutation tests, which randomly shuffles *sample class* labels and computes the probability that the frequency of a given *class* doesn’t occur by random chance. Class samples with *P*<0.05 were considered statistically significant and enriched for the group it was identified in (either the U or M groups).

### Quantitative Reverse-transcriptase PCR (qRT-PCR)

cDNA was synthesized using 100 ng of total RNA in a 20 µl reaction volume. The Superscript® III First Strand synthesis system (Life Technologies, Carlsbad, CA) was used with the following conditions: 10 minutes at 25°C, 30 minutes at 50°C, 5 minutes at 85°C and 20 minutes at 37°C with RNase H. QPCR was subsequently performed on 10 ng of cDNA in a final volume of 25 µl using the ABI 7900HT Fast (Life Technologies, Grand Island, NY). SYBR green fluorescence was used for the detection of amplification after each cycle. Negative (no template) controls were run in parallel to confirm the absence of nonspecific fluorescence in samples. Real-time qPCR was done using the following protocol with Platinum® SYBR® Green qPCR SuperMix (Life Technologies): 2 minutes at 95°C for activation of Platinum® *Taq* DNA polymerase, 15 seconds at 95°C, 30 seconds at 59°C, and 30 seconds at 72°C for 40 cycles. Quantification was based on the number of cycles necessary to produce a detectable amount of product above background. To ensure specificity of the PCR product, the melting curves for the sample products were analyzed. The following QuantiTect Primer assays were purchased (Qiagen, Valencia, CA) for PCR: Hs_DLC1_1_SG (QT00026915); Hs_IGF1R_1SG (QT00005831); Hs_CDKN2A_1_SG (QT00089964); and Hs_IL17RB_1_SG (QT00025956). B-actin was used as an internal reference control. The quantity of expression is calculated relative to the sample with the lowest mean delta (Δ) Ct value for the gene of interest. The equation used for relative fold-change was 2^-ΔΔCT^_._

### Bisulfite Pyrosequencing Analysis

For DNA methylation analysis using pyrosequencing technology, 500 ng of DNA was bisulfite treated (EZ DNA Methylation kit, Zymo Research, Irvine, CA). PCR and sequencing primers for the genes DLC1, IL17RB, CDKN2A and IGF1R were designed with the PyroMark Assay Design software 2.0 (Qiagen, Valencia, CA) by EpigenDX (Hopkinton, MA). Primer sequences for pyrosequencing can be purchased directly from EpigenDX. Assay design details can be found in **Table 1**. PCR was first performed in a thermocycler (Bio-Rad, Hercules, CA) with 0.2 µmol/L of each primer using the following protocol: 2 minutes at 95°C for activation of Platinum® *Taq* DNA polymerase, 30 seconds at 95°C, 30 seconds at 56°C, 30 seconds at 72°C for 45 cycles and 5 minutes at 72°C. One of the PCR primers for each pair was biotinylated for purification of PCR products on Sepharose beads (Amersham Biosciences, Piscataway, NJ). The Sepharose beads containing the immobilized PCR product were washed and denatured using a 0.2 mol/L NaOH solution as recommended by the manufacturer. Subsequently, 0.2 µmol/L pyrosequencing primers were annealed to the purified single-stranded PCR product and PCR products were sequenced using the PyroMark MD System (Qiagen) as per the manufacturer’s instructions. The methylation status of each locus was analyzed as a T/C SNP using QCpG software and the percent methylation for each locus was analyzed. Pyrosequencing interrogated 7, 14, 32, 10 CpG sites for DLC1, p16, IGF1R and IL17RB respectively (**Table 1**). Data is presented as an average of all loci analyzed.

**Table 1:** Summary of assay details for CpG sites interrogated by bisulfite pyrosequencing.

Gene symbol	Assay ID	Assay location	TSS (bp)	Genomic locus (GRCh37/hg19)	CpG island	Number of CpGs
**DLC1**	ADS1077FS2	5′UTR	143 to 218	chr8:13372253-13372178	No	4
**DLC1**	ADS2121FS	Promoter	−97 to −59	chr8:13372492-13372454	No	3
**IGF1R**	ADS2177FS1	5′UTR	104 to 159	chr15:99192303-99192358	Yes	11
**IGF1R**	ADS2177FS2	5′UTR	166 to 241	chr15:99192365-99192440	Yes	15
**IGF1R**	ADS2122re	Intron 1	722 to 780	chr15:99192921-99192979	Yes	8
**IL17RB**	ADS2166FS2	Promoter	104 to 177	chr3:53880710-53880783	Yes	10
**p16**	ADS1193FS1	Promoter	−240 to −207	chr9:21975337-21975304	Yes	3
**p16**	ADS1193FS2	Promoter	−209 to −183	chr9:21975306-21975280	Yes	6
**p16**	ADS1067FS1	5′UTR	208 to 232	chr9:21974890-21974866	Yes	7

## Results

### Identification of Methylation-expression Correlations as Computed by Pearson Correlation

To identify DNA methylation events that correlate to gene expression levels we first computed a Pearson correlation coefficient in a cohort size consisting of 193 MM samples. For this we used 1,505 probes on the methylation BeadArray and the Affymetrix gene expression probe sets that mapped to the same gene. A negative correlation was defined when the directionality of change for expression and methylation were in the opposite direction (e.g. presence of methylation and loss of expression, or vice versa). A positive correlation occurred when the directionality of changes was the same between methylation and expression (e.g. presence of methylation and positive expression, or vice versa). Only 31 (2.1%) CpG loci (corresponding to 24 unique genes) had at least one gene expression probe with a statistically significant correlation, where 19 loci had negative correlations, and the other 12 had positive correlations **(** **Table 2** **)**. These 31 loci showed correlation coefficients ranging from |0.297| to |0.593|. Based on the genes of the GoldenGate methylation array, our data suggests that DNA methylation in MM is, therefore, not strongly associated with gene expression.

**Table 2:** CpG loci with statistically significant methylation-expression relationships as determined by Pearson correlation.

Probe ID	Gene symbol	Correlation coefficient*	CpG island	TSS (bp)
**CHFR_P501_F**	CHFR	−0.404	Yes	−501
**DAPK1_E46_R**	DAPK1	−0.399	Yes	46
**DAPK1_P10_F**	DAPK1	−0.385	Yes	−10
**FRZB_E186_R**	FRZB	−0.375	Yes	186
**GPX1_P194_F**	GPX1	−0.587	Yes	−194
**GPX1_E46_R**	GPX1	−0.405	Yes	46
**GSTP1_E322_R**	GSTP1	−0.593	Yes	322
**HIF1A_P488_F**	HIF1A	−0.417	Yes	−488
**IGF1R_P325_R**	IGF1R	−0.428	Yes	−325
**IGSF4_P86_R**	IGSF4	−0.45	Yes	−86
**IGSF4_P454_F**	IGSF4	−0.405	Yes	−454
**IMPACT_P186_F**	IMPACT	−0.375	Yes	−186
**MEST_E150_F**	MEST	−0.383	Yes	150
**MGMT_P281_F**	MGMT	−0.545	Yes	−281
**MGMT_P272_R**	MGMT	−0.453	Yes	−272
**P2RX7_P119_R**	P2RX7	−0.438	No	−119
**SPARC_E50_R**	SPARC	−0.387	Yes	50
**TJP1_P326_R**	TJP1	−0.488	Yes	−326
**TJP1_P390_F**	TJP1	−0.42	Yes	−390
**CASP3_P420_R**	CASP3	0.327	Yes	147
**CYP2E1_E53_R**	CYP2E1	0.374	No	53
**DLC1_E276_F**	DLC1	0.322	No	276
**DLC1_P695_F**	DLC1	0.495	No	−695
**GLI3_E148_R**	GLI3	0.297	No	148
**HLA-DPB1_P540_F**	HLA-DPB1	0.389	No	−540
**HOXA5_P1324_F**	HOXA5	0.339	Yes	−1324
**HOXA5_P479_F**	HOXA5	0.5	Yes	−479
**PADI4_P1011_R**	PADI4	0.343	No	−1011
**S100A12_P1221_R**	S100A12	0.458	No	−1221
**S100A4_P887_R**	S100A4	0.385	No	−887
**SNRPN_P230_R**	SNRPN	0.313	No	−230

* Average correlation is shown for loci with more than one gene expression probe.

We then examined if the 31 correlated loci were specifically associated with CpG islands or non-CpG islands by examining the average distance to transcriptional start site (TSS) as per the annotation of probes on the GoldenGate array. We found that the 31 CpG sites were generally not associated with CpG islands (*P* = 0.661) and similarly, only 3 of 12 probes showing positive correlations were located within CpG islands (*P* = 1.000, **Table 3**). Positively correlated loci were, on the other hand, significantly associated with non-CpG islands (*P* = 0.002). In contrast, of 19 methylation probes showing negative correlations, 18 were associated with CpG islands (*P* = 0.009). Loci with positive correlations were, in general, 480 base pairs (bp) upstream of the TSS compared with negatively correlated methylation probes, which on average were 149 bp upstream of the TSS **(** **Table 3** **)**.

**Table 3:** Relationship to CpG islands and TSS for genes with methylation-expression correlations identified by computing a Pearson correlation coefficient.

	Number of probes	CpG island	Non-CpGisland	*P-value* * for CpG island	*P-value* * for non-CpG island	Average TSS	Median TSS
**All methylation loci**	1,505	1,044	461	N/A	N/A	−227.1	−165
**Loci with correlation**	31	21	10	0.661	0.489	−277.3	−230
**Loci with positive correlation**	12	3	9	1.000	**0.002**	−480.3	−509.5
**Loci with negative correlation**	19	18	1	**0.009**	0.999	−149.1	−186.0

* Hypergeometric *P* values are given.

### Identification of Methylation-expression Correlations Using a Discretization Approach

In addition to the genes with statistically significant Pearson correlation coefficients, we examined methylation-expression correlations using a non-linear approach. This is referred to as a *discretization approach* because samples are categorized into different groups according to their methylation levels for each probe on the methylation BeadArray **(** **Figure 1B** **)**. Samples with one standard deviation above or below the mean for any given probe were considered *methylated* (M) or *unmethylated* (U), respectively. Statistically significant gene expression differences between samples in the *M* and *U* groups would suggest a methylation-expression correlation at a given locus. By applying this method, we identified 382 CpG loci (25.3%, 309 unique genes) with methylation-expression correlations. When averaged across 382 probes, there were 30 samples in the *M* groups and 27 samples in the *U* groups. Of 382 loci, 113 loci had positive correlations and 269 loci had negative correlations. The top 40 correlations using the discretization method are shown in **Table 4**. This included the top 20 probes with positive correlations and the top 20 probes with negative correlations. We also included an additional 8 probes that were common to genes identified by Pearson correlation but did not rank in the top 40.

**Table 4:** Top 40 methylation loci with statistically significant methylation-expression correlations using the *discretization approach*.

Probe ID	Gene symbol	CpGisland	TSS (bp)	Direction of correlation*	Sample class enrichment in *methylated* samples (*P-value*)†	Sample class enrichment in *unmethylated* samples (*P-value*)†
**CAV2_E33_R**	CAV2	Yes	33	−	None	None
**CCND1_E280_R**	CCND1	Yes	280	−	1q amp (0.0028), D2 (0.0248)	11q13 (0.0322)
**CHFR_P501_F**	CHFR	Yes	−501	−	13 monosomy (0.0343), 1q amp (0.0112), D2 (0.0031)	None
**DAPK1_E46_R**	DAPK1	Yes	46	−	D2 (0.0424)	H (0.0176), D1 (0.0360)
**DNASE1L1_P39_R**	DNASE1L1	Yes	−39	−	D1+D2 (0.0365)	H (0.0446)
**(FRZB_E186_R)**	FRZB§	Yes	186	−	4p16 (0.0045)	11q13 (0.0411)
**GPX1_P194_F**	GPX1	Yes	−194	−	NH (0.0069), 1qAmp (0.0335), 11q13 (0.0039), 4p16 (0.0004)	D1 (0.0389)
**GSTM2_P109_R**	GSTM2	No	−109	−	None	None
**GSTP1_E322_R**	GSTP1	Yes	322	−	H (0.0026), D1 (0.0031)	None
**(HIF1A_P488_F)**	HIF1A	Yes	−488	−	None	13 monosomy (0.0028), 4p16 (0.0016)
**IGF1R_E186_R**	IGF1R‡	Yes	186	−	H (0.0014), D1 (0.0003)	NH (0.0049), 4p16 (0.0024)
**IGF1R_P325_R**	IGF1R‡	Yes	−325	−	H (0.0004), D1 (0.0002)	11q13 (0.0401)
**IGSF4_P86_R**	IGSF4	Yes	−86	−	None	H (0.0259)
**(IGSF4_P454_F)**	IGSF4	Yes	−454	−	D1 (0.0370)	17p del (0.0330)
**IL17RB_E164_R**	IL17RB‡	Yes	−560	−	H (0.0058)	NH (0.0353), 11q13 (0.0103), Maf (0.0172)
**(IMPACT_P186_F)**	IMPACT	Yes	−186	−	1q amp (0.0419)	None
**(MEST_E150_F)**	MEST	Yes	150	−	None	None
**MGMT_P281_F**	MGMT	Yes	−281	−	None	None
**MGMT_P272_R**	MGMT	Yes	−272	−	None	None
**(P2RX7_P119_R)**	P2RX7	No	−119	−	H (<0.0001), D2 (0.0207)	None
**PTK2_P735_R**	PTK2	Yes	−735	−	D1 (0.0328)	None
**ROR2_E112_F**	ROR2	Yes	112	−	None	None
**SFN_E118_F**	SFN	Yes	118	−	None	NH (0.0016), 17p del (0.0151)
**(SPARC_E50_R)**	SPARC	Yes	50	−	H (0.0311)	1q amp (0.0399)
**TGFA_P642_R**	TGFA	Yes	−642	−	None	None
**TJP1_P326_R**	TJP1	Yes	−326	−	None	None
**(TJP1_P390_F)**	TJP1	Yes	−390	−	None	None
**VBP1_P194_F**	VBP1	Yes	−194	−	D1+D2 (0.0414)	17p del (<0.0001)
**ACVR1B_E497_R**	ACVR1B	Yes	497	+	None	None
**CASP3_P420_R**	CASP3	Yes	147	+	None	D2 (0.0317)
**CDH17_P376_F**	CDH17	No	−376	+	D1+D2 (0.0446)	1q amp (0.0371)
**DLC1_P695_F**	DLC1‡	No	−695	+	H (<0.0001), D1 (0.0014)	NH (0.0001), 1q amp (0.0121), 11q13 (<0.0001)
**DLC1_E276_F**	DLC1‡	No	276	+	H (0.0280), D1 (0.0265)	NH (0.0209), 11q13 (0.0265)
**DLC1_P88_R**	DLC1‡	No	−88	+	13 monosomy (0.0113), 1q amp (0.0072), 4p16 (<0.0001)	NH (0.0403)
**FRZB_P406_F**	FRZB§	Yes	−406	+	6p21 (0.0435)	None
**HLA-DPB1_P540_F**	HLA-DPB1	No	−540	+	None	11q13 (0.0226)
**HOXA5_P1324_F**	HOXA5	Yes	−1324	+	H (0.0466)	None
**HOXA5_P479_F**	HOXA5	Yes	−479	+	H (0.0171)	4p16 (0.0030)
**ITPR2_P804_F**	ITPR2	Yes	−804	+	1q amp (0.0338)	13 monosomy (0.0260)
**p16_seq_47_S188_R**	p16‡	Yes	188	+	H (0.0216), 13 monosomy(0.0278), D1 (0.0348)	NH (0.0018), Maf (0.0016)
**p16_seq_47_S85_F**	p16‡	Yes	−85	+	H (0.0289), D1 (0.0332)	None
**PADI4_P1011_R**	PADI4	No	−1011	+	None	None
**S100A12_P1221_R**	S100A12	No	−1221	+	4p16 (<0.0001)	1q amp (0.0338), 17p del (0.0364), 11q13 (0.0350)
**SLC6A8_P409_F**	SLC6A8	Yes	−409	+	None	None
**SNRPN_P230_R**	SNRPN	No	−230	+	4p16 (0.0029)	13 monosomy (0.0314), 1q amp (0.0204), D2 (0.0025)
**THBS1_P500_F**	THBS1	Yes	−500	+	None	None
**TNFRSF10C_P612_R**	TNFRSF10C	No	−612	+	None	H (0.0292), 17p del (0.0154), D2 (0.0020)
**WNT10B_P993_F**	WNT10B	Yes	−993	+	NH (0.0114)	None

Additionally, loci in common with the Pearson correlation method are shown in parentheses.

* (−) indicates a negative methylation-expression correlation, where high methylation correlates to lower expression or vice versa. (+) indicates a positive correlation where high methylation correlates to increased expression or vice versa.

† P values were obtained after 10,000 random permutations of each class label.

‡ Gene was selected for further validation.

§ The methylation level of the probe FRZB_P406_F has a positive correlation with gene expression by both Pearson correlation and the discretization method, while the other probe FRZB_E186_R has a negative methylation-expression correlation by both methods. Only the FRZB_P406_F probe is shown for the discretization method due to its statistical significance.

We also examined whether CpG loci were located in CpG islands or non-CpG islands **(** **Table 5** **)**. Consistent with the Pearson method above, the discretization approach demonstrated that loci with positive correlations were significantly associated with non-CpG islands (*P* = 9.26×10^−6^) and loci with negative correlations were significantly associated with CpG islands (*P* = 3.29×10^−6^).

**Table 5:** Relationship to CpG islands and TSS for genes with methylation-expression correlations identified by the *discretization approach*.

	Number of probes	CpGisland	Non-CpGisland	*P-value* * for CpG island	*P-value* * for non-CpG island	Average TSS	Median TSS
**All methylation loci**	1,505	1,044	461	N/A	N/A	−227.1	−165
**Loci with correlation**	382	274	108	0.137	0.890	−222.5	−153
**Loci with positive** **correlation**	113	57	56	1.000	**9.26E-6**	−282.7	−186.0
**Loci with negative correlation**	269	217	52	**3.29E-6**	1.000	−197.2	−151.0

* Hypergeometric *P* values are given.

**Figure 2:**
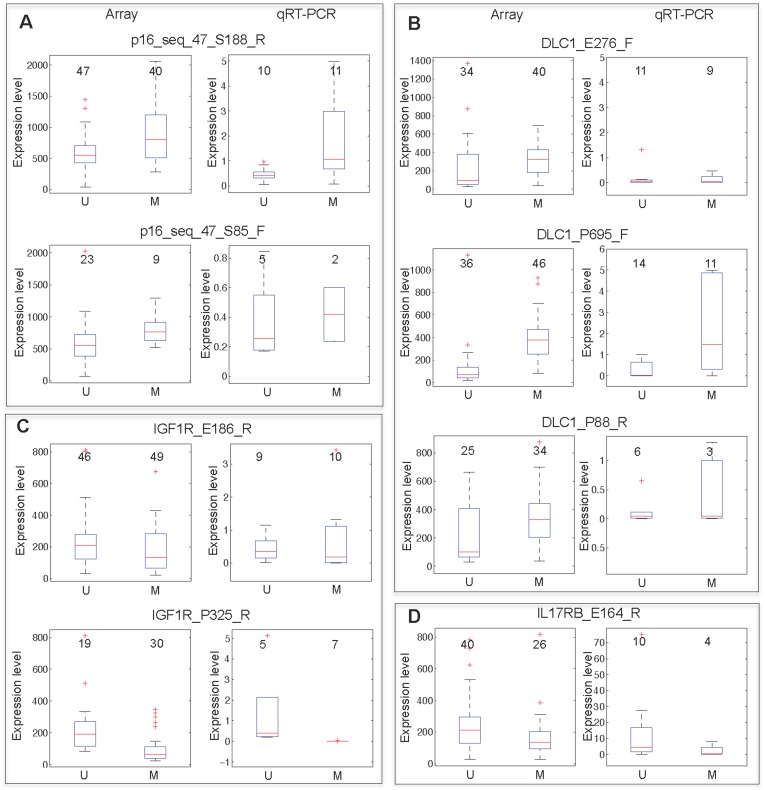
Confirming expression trends by qRT-PCR for correlated genes identified by *discretization approach*. Box plots represent gene expression levels generated by either microarray or qRT-PCR. Data are shown for samples classified as U or M based on the methylation status of p16 (A), DLC1 (B), IGF1R (C), or IL17RB (D). For microarray data, probe intensities are plotted on the y-axis. Relative fold-change differences are plotted for data generated by qRT-PCR. The number of samples in each group is displayed above each plot. The GoldenGate BeadArray probe names are indicated above each pair of box plots.

Among the correlated genes were DLC1, p16, IGF1R and IL17RB. Only DLC1 and IGF1R were identified by both Pearson correlation and discretization methods. The genes p16 and DLC1 were methylated (based on two and three CpG probes respectively) and expressed (positive correlation), while IGF1R and IL17RB were unmethylated (based on two and one CpG probes respectively) and expressed (negative correlation). To validate the expression relationship for each of these genes with methylation we performed qRT-PCR analysis on 46 samples selected randomly as a subset of the 193 MM samples used in the study. The results of the gene expression array for each of the four genes were compared against the qRT-PCR data for M and U groups. A positive methylation-expression correlation was confirmed for each methylation probe representing p16 **(** **Figure 2A** **)** and DLC1 **(** **Figure 2B** **)**. Similarly, a negative correlation was confirmed for IGF1R **(** **Figure 2C** **)** and IL17RB **(** **Figure 2D** **)**. Differences in expression between U and M groups were statistically significant (*P*<0.05, by t-test).

### Sample Class Analysis Reveals Enrichments of Chromosomal Aberrations and TC Class for Samples Classified as *methylated* and *unmethylated* for Different Genes

**Figure 3:**
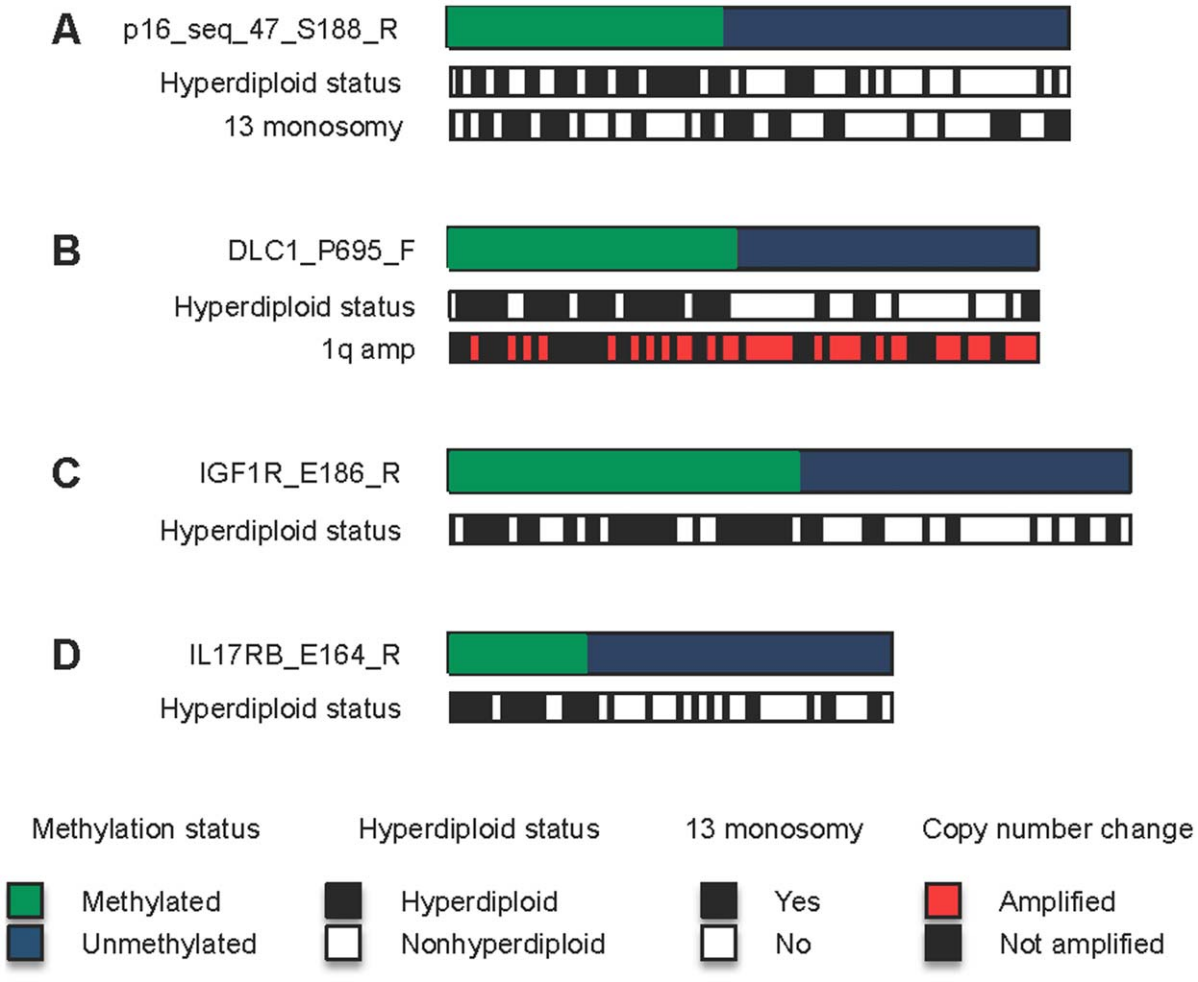
A diagrammatic representation of *sample class* enrichments for selected genes. Samples classified as M or U by the discretization approach are indicated by green and blue bars respectively. The genomic context of samples in each group are shown as white, black or red bars. A representative locus is shown for p16 (A), DLC1 (B), IGF1R (C) and IL17RB (D).

We performed a molecular class enrichment analysis to identify genomic characteristics associated with samples in both the *methylated* and *unmethylated* sample groups for each of the 382 methylation probes identified above. The data are presented in **Table 4**. Higher methylation levels of p16 (2 CpG probes), IGF1R, IL17RB and DLC1 (2 of 3 CpG probes) were associated with hyperdiploid MM **(** **Figure 3** **,** **Table 4** **)**. Other enrichments included 13 monosomy and 1q gain for p16 and DLC1 genes **(** **Table 4** **)**. The 4p16 TC class was also associated with samples exhibiting higher levels of DLC1 methylation **(** **Table 4** **)**. In contrast, low-level methylation of p16 (1 of 2 probes), IGF1R (1 of 2 probes), DLC1 or IL17RB was strongly associated with nonhyperdiploid MM. In addition, low-level methylation (U groups) showed enrichments for 1q gain (DLC1), 11q13 molecular class (IGF1R, IL17RB, and DLC1) or 4p16 (IGF1R). It is important to note that the sample combination that makes up U and M groups are different even for probes belonging to the same gene. This accounts for the different class enrichments observed within different probes representing one gene. Representative sample class enrichment patterns for these four genes are shown in **Figure 3** **.**

### Comparison of Methods for Methylation-expression Correlated Genes

**Figure 4:**
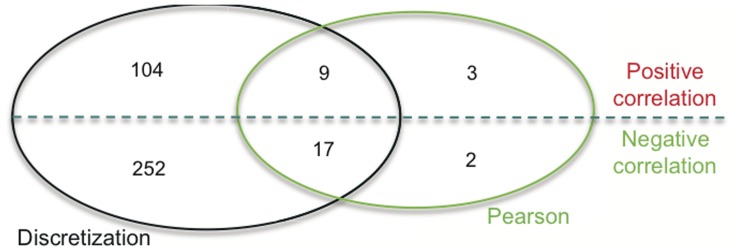
Comparison of Pearson and discretization approaches used to identify methylation-expression correlations. The Venn Diagram displays the total number of overlapping loci with positive/negative methylation-expression correlations identified by computing either a Pearson correlation or applying the discretization method.

Next we examined the degree of overlap between the two methods used to determine methylation-expression correlations. Of 31 CpG loci detected by computing a Pearson correlation coefficient, 26 were also detected by the discretization approach. The five CpG probes that were identified only by Pearson correlation were the following: CYP2E1_E53_R, DAPK1_P10_F, GLI3_E148_R, GPX1_E46_R, and S100A4_P887_R. When we examined these five loci unique only to the Pearson method, we discovered that the methylation values for those genes were clustered to one end of the methylation scale. This explained their exclusion from the discretization approach, which relies on methylation values falling on both low and high ends of the methylation spectrum. Of the 26 common loci, the direction of correlation was always the same. There were 17 negatively correlated and 9 positively correlated loci in common to the two analysis methods **(** **Figure 4** **)**.

### Validating Methylation-expression Correlations of Selected Genes by Bisulfite Pyrosequencing and qRT-PCR in an Independent Cohort

In order to confirm the reliability of our findings we obtained 43 additional MM patient samples and performed bisulfite pyrosequencing and qRT-PCR to analyze DLC1, p16, IGF1R and IL17RB for CpG methylation and expression respectively. These samples were used as part of MMRC Genomics Initiative but were not included as part of the 193 samples used to make the original observations, and thus constitute an independent validation sample cohort. Percent methylation (average of all CpG loci interrogated by pyrosequencing) and relative expression fold-change values were log2-transformed. We interrogated a total of 7, 14, 32, and 10 CpG sites for DLC1, p16, IGF1R and IL17RB respectively. These loci covered many more loci then interrogated by the GoldenGate array and were clustered in two different genic regions **(** **Table 1** **)**. Next we computed a Pearson correlation coefficient and applied the discretization approach to the dataset as described above to confirm the relationship of methylation to gene expression for the selected genes.

**Figure 5:**
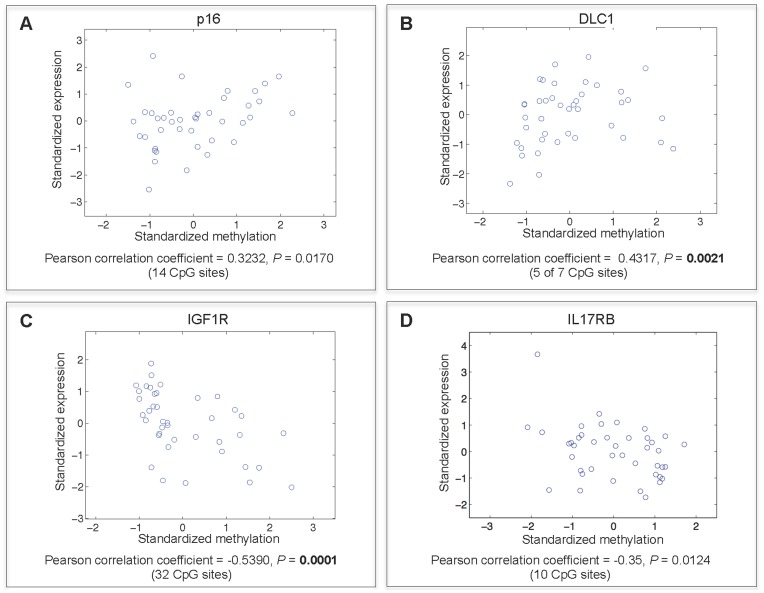
Validation of methylation-expression relationships by Pearson correlation for p16, DLC1, IGF1R and IL17RB using pyrosequencing and qRT-PCR in an independent sample set. Scatter plots depict the extent of linear correlation of DNA methylation (x axis) to gene expression (y axis) for p16 (A), DLC1 (B), IGF1R (C), IL17RB (D) in 43 MM samples. Methylation data was generated by bisulfite pyrosequencing and the average percent methylation for all CpG loci interrogated is shown. Gene expression relative fold-change was obtained by qRT-PCR. Pearson correlation coefficients are shown and *P* values are generated by random permutation tests.

**Figure 6:**
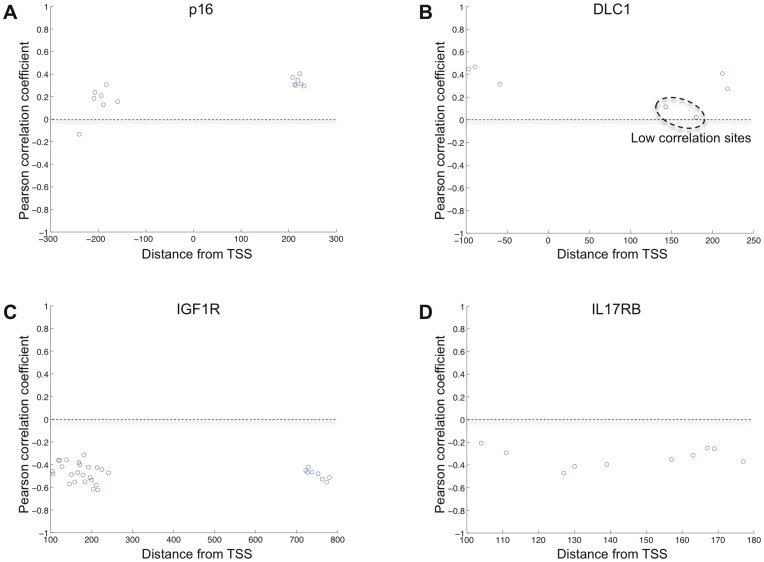
Pearson correlation coefficients calculated for methylation of individual CpG loci. Graphs show the Pearson correlation coefficient (y-axis) of individual CpG loci analyzed by pyrosequencing to the expression level for the corresponding gene. The distance to TSS is shown (x-axis) to demonstrate the proximity of each CpG locus to each other. Each CpG locus analyzed for p16 (A), DLC1 (B), IGF1R (C) and IL17RB (D) is depicted as a point on the graph.

Computing a Pearson correlation coefficient demonstrated positive methylation-expression trends for DLC1 and p16 and negative correlation trends for IGF1R and IL17RB (**Figure 5** **,** **6**). Similar to the original analysis **(** **Table 2** **)**, the validation analysis demonstrated statistically significant Pearson correlations only for IGF1R and DLC1. IGF1R had significant Pearson correlation coefficients (*ρ* = −0.5390, *P* = 0.0001) based on the analysis of 8 CpG sites located about +700–+800 bp of the TSS and 24 CpG sites located about +100–+250 bp of the TSS **(** **Figure 5C** **and** **6C** **)**. For DLC1, average methylation of seven CpG sites analyzed by pyrosequencing demonstrated a low correlation (*ρ* = 0.1974, *P* = 0.1002), which was due mainly to two CpG loci **(circled in** **Figure 6B** **)** located in a non-CpG island region +143 and +180 bp of the TSS. When these loci were excluded, DLC1 also showed statistically significant correlation (*ρ* = 0.4317, *P* = 0.0021) based on the remaining 5 CpG loci **(** **Figure 5B** **)**. The reasons for this are unclear but is suggestive that CpG loci, particularly those in non-island regions, can have site-specific regulatory effects.

**Figure 7:**
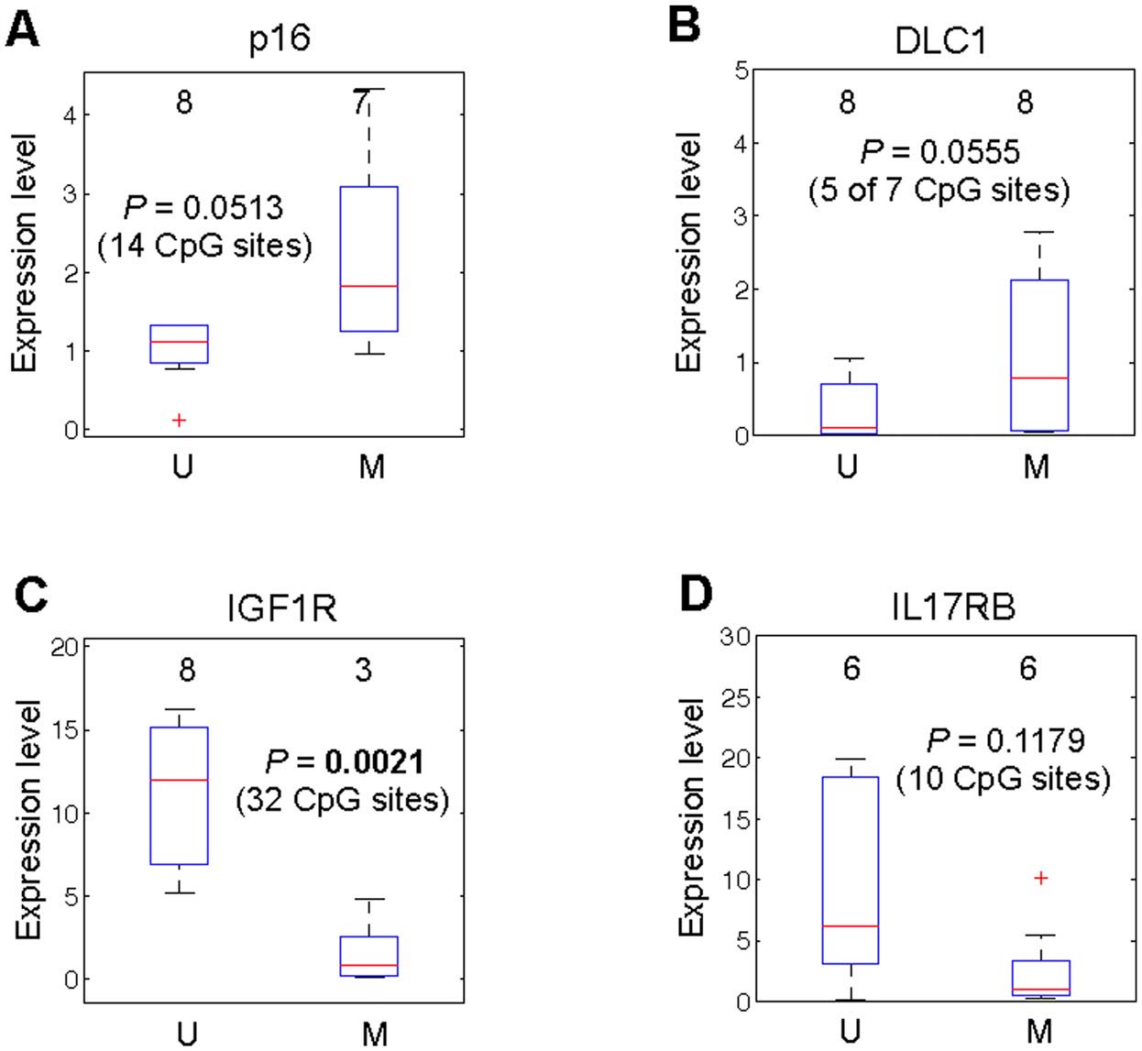
Validation of methylation-expression relationships using a discretization approach for p16, DLC1, IGF1R and IL17RB by pyrosequencing and qRT-PCR in an independent sample set. Bisulfite pyrosequencing data for 43 independent samples was used to discretize samples into methylated (M) or unmethylated (U) groups based on the percent methylation values obtained for p16 (A), DLC1 (B), IGF1R (C) and IL17RB (D). The average of all CpG loci interrogated by pryosequencing for each gene was used in the analysis. Differential gene expression analysis was conducted by qRT-PCR to compare the expression of each gene between U and M groups. Positive methylation-expression correlations were confirmed for p16 and DLC1. A negative correlation was validated for IGF1R and IL17RB. The number of patients in U and M groups is given above each box plot. Y-axis represents expression levels by plotting relative fold-change (2^−ΔΔCT^).

Next we applied the discretization approach by categorizing the 43 samples into M and U categories based on the mean and standard deviation of percent methylation values and then compared the gene expression differences between the U and M groups as described above. Consistent with our array-based findings, p16 (**Figure 7A**) and DLC1 (**Figure 7B**) demonstrated positive methylation-expression tendencies. IGF1R (**Figure 7C**) and IL17RB (**Figure 7D**) demonstrated negative correlations, as expected. However, this analysis led to statistical significance only when U and M groups were compared for IGF1R (*P* = 0.0021). The other three genes, while demonstrated expected trends, did not reach a significant P value (*P*<0.05) but by only a small margin in most cases **(** **Figure 7** **)**. We feel this is related to the small number of samples that ultimately comprised U and M groups **(** **Figure 7** **)**.

### Determining the Frequency of Differential CpG Methylation in MM

In order to further examine the low number of methylation-expression correlations in MM as identified by the GoldenGate methylation array, we determined the frequency of differential CpG methylation 193 MM samples assayed. We conducted a differential methylation analysis by comparing 193 MM samples to NPC as previously described [13]. We identified 222 differentially methylated loci. Of these, 186 were hypomethylated. We found that 92% of samples displayed hypomethylation in at least 50% of hypomethylated CpG loci on the array and 52% displayed hypomethylation in at least 75% of probes. These data indicate that there is a disproportionately higher frequency of CpG hypomethylation than there is altered expression for the genes examined; further supporting the notion that methylation is poorly correlated to gene expression.

## Discussion

The aberrant epigenetic landscape of a cancer cell is characterized by global genomic hypomethylation, CpG island promoter hypermethylation of tumor suppressor genes, changes in histone modification patterns, altered nucleosome positioning, as well as altered expression profiles of chromatin-modifying enzymes [10], [11], [18]. The purpose of the current study was to determine the association of DNA methylation to gene expression in MM. Typically, DNA hypermethylation at promoter regions represents a mechanism of transcriptional silencing, while a decrease in DNA 5-methylcytosine may ultimately facilitate the aberrant expression of proto-oncogenes [19], [20], [21]. More recently, studies have also shown that when genes are active, their CpG island promoters are situated in nucleosome-depleted regions [11], [18].

The findings of our study demonstrated that DNA methylation was, in general, weakly associated with gene expression when specific loci representing about 800 genes were examined. Reasons for the lack of correlation could include that 5-methylcytosine may be necessary but not sufficient for regulating gene expression and that the nature of chromatin formed on a methylated template is what renders it transcriptionally active or inactive. Moreover, researchers in the field are now suggesting that DNA methylation may not be the predominant pathway for silencing genes [18]. 5-methylcytosine must be removed by either passive or active mechanisms to establish a permissive state for subsequent gene expression and it is not always clear whether methylation changes are a result of transcription or whether they stabilize transcriptionally incompetent states [18].

Nevertheless, we identified a subset of genes that were either negatively or positively correlated to DNA methylation. For this subset of genes, genes with inverse methylation-expression correlations were generally situated within CpG islands upstream of TSS and those with positive correlations were associated with non-CpG islands. Our data is consistent with other genome-wide studies of the methylome, which emphasized that the position of methylation influences its relationship to gene expression [14], [15]. For example, methylation at CpG sites, in the vicinity of TSS, or located at the edges or “shores,” of promoter-associated CpG islands has been inversely correlated with gene expression [15]. Methylation in gene bodies, which are mostly CpG-poor, does not block and might even stimulate transcription elongation, and may impact splicing [14], [15], [18].

To validate the presence of both negative and positive methylation-expression correlations we selected four genes for further study. DLC1 and p16 showed a positive methylation-expression correlation (based on the methylation levels of seven and 14 CpG sites respectively), and IGF1R and IL17RB both had a negative correlation (based on the methylation levels of 32 and 10 CpG sites respectively). DLC1 (deleted in liver cancer 1), a tumor suppressor gene that encodes a RhoGTPase-activating protein, is recurrently downregulated or silenced in various solid tumors and hematological malignancies because of epigenetic modifications or genomic deletion [22], [23]. In MM, previous studies have shown methylation and inactivation of DLC1 in a high frequency of myeloma cell lines [22], [23]. The same group also reported hypermethylation of DLC1 in 11 of 14 primary MM samples but without mention of the gene expression levels [22], [23]. Deregulation of DLC1 by DNA methylation is likely to be important in the pathogenesis of multiple myeloma by altering signaling associated with Rho-GTPases, which impacts cytoskeletal architecture and cellular motility. Indeed, re-expression of DLC1 in MM has been shown to inhibit myeloma cell migration [22], [23]. The tumor suppressor gene p16 is known to be frequently hypermethylated in MM [24], [25], [26], [27]. Several reports from our labs and others have shown that the frequency of p16 hypermethylation increases with the progression of MM [24], [25], [28] without affecting gene expression levels [25].

Hypomethylated genes that are inversely correlated to gene expression are of particular interest and could represent potential oncogenes. Among these include IGF1R (Insulin-like growth factor receptor 1) and IL17RB (Interleukin-17 receptor B). Although little is known regarding the role of IL17RB in MM, a recent study observed that IL-17 produced by TH17 cells promotes MM cell growth, colony formation and tumor growth *in vivo* via IL17R [29]. Thus, additional studies around IL17RB are warranted to determine whether IL17RB can be used for therapeutic targeting in MM. IGF1R, which demonstrated the strongest correlation of the four genes, is a transmembrane tyrosine kinase that is frequently overexpressed in tumors including MM. IGF1R-methylated samples (based on the methylation levels of two CpG probes) tended to be hyperdiploid, and those samples with low levels of methylation were associated with the 4p16 TC class, which is a poor prognostic factor in MM. Our class enrichment analysis for IGF1R confirms findings demonstrating that overexpression of IGF1R is associated with the 4p16 TC class [30]. Insulin growth factor-1 (IGF-1) is an important survival and growth factor in MM and various other malignancies [31], [32], [33]. Recent efforts have shown *in vitro* and clinical efficacy of targeting IGF1R in MM using small molecule inhibitors or a humanized anti-IGF1R monoclonal antibody [34], [35], [36], [37]. Our study lends further evidence to the potential importance of IGF1R as a therapeutic target in MM.

In summary our data demonstrates that DNA methylation is poorly correlated to the expression of approximately 800 genes when specific loci were examined. Other methylation platforms, such as the 450 K Infininum array by Illumina and MethylC-seq, were not available at the time we performed our methylation studies so we were not able to extend our findings beyond the ∼800 gene set or to cover a larger number CpG loci per gene. Still, however, the genes on the GoldenGate array represent many of the most relevant cancer-associated genes. Furthermore, our validation studies examined many more CpG loci beyond those interrogated by the array. Undoubtedly, more studies are needed to fully appreciate methylation-expression relationships in MM. In our study we also identified a number of interesting class enrichments, whereby a genetic characteristic was linked to the methylation (or lack of ) of a particular CpG locus. These enrichments often differed for different loci within the same gene. While we currently don’t understand the scope of these findings, it is possible that site-specific methylation can be preferential to tumors with certain genetic features and can alter the biology of patients of different subtypes in different ways. Additionally, exploring the reasons why low and high methylation levels of genes such as DLC1, p16, IGF1R and IL17RB were associated with non-hyperdiploid or hyperdiploid MM respectively are among the findings that warrant further investigation. The identification of these clinically-pertinent class enrichments warrant future studies to examine the biological and prognostic relevance of site-specific CpG locus methylation and its relationship to tumor class. Discerning the spectrum of DNA methylation functions will undoubtedly be essential for further understanding myelomagenesis and for developing strategies and novel drugs to target the epigenome in MM.
